# Modeling and Simulation of Eddy Current Dissipation Magnetic Acceleration Noise of Space Inertial Sensors

**DOI:** 10.3390/s24237723

**Published:** 2024-12-03

**Authors:** Pengxuan Li, Zhiyin Sun, Wei Gao, Bingzhang Cao, Yunzhao Li, Liyi Li, Lei Wang

**Affiliations:** 1 School of Instrumentation Science and Engineering, Harbin Institute of Technology, Harbin 150001, China; pengxuan_li@126.com (P.L.); caobingzhang@stu.hit.edu.cn (B.C.); 2 School of Electrical Engineering and Automation, Harbin Institute of Technology, Harbin 150001, China; sunzhiyin@hit.edu.cn (Z.S.); 23b906035@stu.hit.edu.cn (Y.L.); liliyi@hit.edu.cn (L.L.); 3 Institute of Disaster Prevention and Reduction Equipment, Institute of Disaster Prevention, Langfang 065201, China; gw_0_0@126.com; 4 Laboratory for Space Environment and Physical Sciences, Harbin Institute of Technology, Harbin 150001, China

**Keywords:** eddy current loss, damping loss, fluctuation-dissipation theorem, magnetic acceleration noise

## Abstract

The magnetic acceleration noise (MAN) that stems from the eddy current dissipation of a test mass (TM) serves as an important source of noise for space inertial sensors. Given the problem that the eddy current dissipation magnetic acceleration noise (ECDMAN) of a cubic TM defies analytical solutions, an analytical model of ECDMAN for a spherical TM, which has the same volume as the cubic TM, is systematically derived on the basis of the principles of electromagnetism and the fluctuation-dissipation theorem, and this model can be used as an approximate analytical model for the evaluation of this noise term. Based on the approximate analytical model, with the TM of the LISA Pathfinder (LPF) as the research object, this paper obtains a modification coefficient using the approach of combining the analytical method with the finite element method (FEM), and establishes a semi-analytical model of ECDMAN for the cubic TM. Using the parameters of the LPF’s TM, the calculation error of the semi-analytical model is reduced by about 4.64% compared with the approximate analytical model. Finally, a generalized modeling approach for the semi-analytical model of ECDMAN is put forward, which is applicable to TMs with different parameters and can realize the real-time and rapid evaluation of ECDMAN during in-orbit experiments.

## 1. Introduction

The space gravitational wave detection mission imposes extremely stringent requirementsregarding on the detection sensitivity of the laser interferometry measurement system [1,2,3]. The TM of the inertial sensor [4,5], functioning as the reflector of the laser interferometer [6,7], supplies a high-precision inertial reference for the laser interferometry measurement system. However, various environmental factors [8,9,10,11,12,13], such as residual gas molecules, electric fields, and magnetic fields, will introduce acceleration noise to the TM, and this turns out to be the crucial factor that restricts the detection sensitivity as well as the detection accuracy of the laser measurement system. Among these environmental factors, the acceleration noise induced by the magnetic field makes up approximately 10~24% of the total noise [14,15,16], and it is an important source of acceleration noise for the inertial sensor. Therefore, exploring the sources of MAN and establishing its analysis model are of great guiding significance for the design of inertial sensors, and are also a crucial link in constructing the index system of the inertial sensor noise.

Considerable research has been carried out by various relevant research teams regarding MAN. The studies reported in [15,16,17] established a measurement method with high resolution for the magnetic characteristics of the TM, based on a precision torsion balance. Diaz-Aguiló et al. proposed an interpolation algorithm for magnetic fields based on neural networks [18]. Armano et al. conducted a statistical analysis of the in-orbit magnetic field data of the LPF to determine the source, magnitude, and influencing factors of the magnetic field [19]. Su et al. adopted the SWMF model and the Tsyganenko model, respectively, to obtain the magnetic field parameters of the TianQin satellite orbit, which provided high-precision parameter inputs for the evaluation of MAN [13,20]. In relation to MAN modeling, the studies in [7,21,22] systematically established a mathematical model of MAN based on the magnetic force formula of a magnetic dipole located in a magnetic field. Sun et al. analyzed the applicable conditions of the above model [23]. Fang et al. took the Taiji mission as the research object, carried out a detailed decomposition and simulation of electromagnetic force noise, and presented a list of electromagnetic parameters that meet the index requirements. The above work is of great significance for the refined modeling, analysis, and evaluation of the MAN of inertial sensors, but there are still some inadequacies. Firstly, all the studies in [12,13,14,15,16,17,18,19,20,21,22,23,24] regard the TM as a magnetic dipole and conduct research based on the magnetic force formula of a magnetic dipole. However, the TM made of the gold-platinum alloy features not only a weak magnetic susceptibility with a value of 2.5 × 10^−5^, but also prominent electrical characteristics with a conductivity of 3.33 × 10^−6^ S/m. These two parameters are isotropic and will induce a series of acceleration noise related to electromagnetic effects, with ECDMAN being one of them. Secondly, although the study in [24] has conducted research on the ECDMAN and presented the model of the disturbance force associated with eddy current damping dissipation, it failed to provide the modeling process. Therefore, the theoretical basis and modeling ideas of the model of ECDMAN are not clear.

Taking into account the drawbacks stated above, in this paper, on the basis of the analytical model of ECDMAN of a spherical TM which has the same volume as the cubic TM, a semi-analytical model is established through an approach that combines the analytical method with the FEM, and a generalized modeling method for ECDMAN is summarized. The semi-analytical model has high computational accuracy and can realize real-time and rapid computational processing of this noise term in future in-orbit experiments.

## 2. Analytical Model

At present, the TM of inertial sensors used for space gravitational wave detection missions all adopt a cubic scheme [25,26,27]. However, in electromagnetics, the induced eddy current of a cubic conductor or a magnetic body cannot be obtained analytically. The rotating ellipsoid is the only geometric shape with finite volume for which the analytical solutions of the induced eddy current can be obtained. The sphere is a special case of the rotating ellipsoid, with its major axis and minor axis being of equal length, meaning that its diameters are equal in all spatial directions. The lengths of the edges of a cube in all spatial dimensions are equal. Therefore, the feature that the lengths of the edges of a cube are equal in all dimensions is most similar to the feature that the diameters of a sphere are equal in all directions. Moreover, the eddy current loss is a volume integral effect. Therefore, a spherical TM with the same volume as the cubic TM is used to study the solution of the ECDMAN.

### 2.1. Eddy Current Loss of Spherical TM

**Figure 1:**
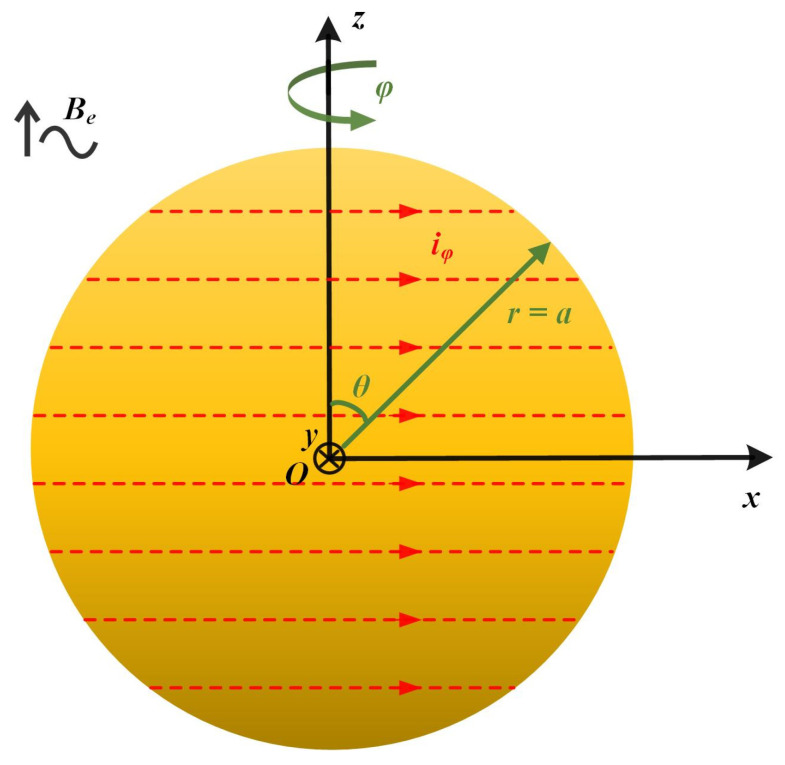
Schematic diagram of the induced eddy current of a spherical TM under the action of AUMF. The red dotted lines represent the eddy current, and the arrows point to their flow direction.

As shown in Figure 1, for the spherical TM, its radius is *a*, the magnetic susceptibility is *χ*, and the electrical conductivity is *σ*. It is assumed that *χ* and *σ* are isotropic and linear within the volume *V’*. Taking the center of the TM as the coordinate origin, a Cartesian coordinate system *O* (*x*, *y*, *z*) and a spherical coordinate system *O* (*r*, *φ*, *θ*) are established. For the convenience of analysis, it is assumed that the sensitive axis direction of the inertial sensor is along the *z*-direction. Under the action of an alternating uniform magnetic field (AUMF) ${\dot{B}}_{e}\;$in the *z*-direction, the TM generates induced eddy current ${\dot{i}}_{\varphi }$. In the spherical coordinate system, ${\dot{i}}_{\varphi }$ flows along the azimuth direction *φ* and the vector magnetic potential $\dot{A}$ only has a component along the *φ* direction. The $\dot{A}$ fulfills the following partial differential equation

(1) $$\left\{\begin{array}{ll} \nabla^{2}\dot{A}=0 & a<r<+\infty \\ \nabla^{2}\dot{A}=\sigma \mu \frac{\partial \dot{A}}{\partial t} & 0<r<a \end{array}\right.$$

Using the method of separation of variables, we find that

(2) $$\dot{A}=\left\{\begin{array}{ll} r^{-1/2}[{\dot{A}}_{n}P_{n}^{1}(\cos\theta )+{\dot{A}}_{n}^{'}Q_{n}^{1}(\cos\theta )][{\dot{B}}_{n}I_{n+1/2}(\lambda r)+{\dot{B}}_{n}^{'}K_{n+1/2}(\lambda r)]e_{\varphi } & 0<r<a\\ ({\dot{C}}_{n}r^{n}+{\dot{C}}_{n}^{'}r^{-n-1})[{\dot{D}}_{n}P_{n}^{1}(\cos\theta )+{\dot{D}}_{n}^{'}Q_{n}^{1}(\cos\theta )]e_{\varphi } & r>a \end{array}\right.$$

In this equation, $\lambda ={\left(j2\pi f\mu \sigma \right)}^{1/2}$, where *j* is the imaginary unit, *μ* represents the magnetic permeability of the TM, and σ denotes the conductivity of the TM. *I_n+_*_1/2_(▪) and *K_n+_*_1/2_ (▪) are the modified Bessel functions of the first kind and the second kind with half-integral order, respectively. $P_{n}^{1}\left(▪\right)$ and $Q_{n}^{1}\left(▪\right)$ represent the associated Legendre functions of the first kind and the second kind, respectively. ${\dot{A}}_{n}$, $A_{n}^{’}$, ${\dot{B}}_{n}$, $B_{n}^{’}$, ${\dot{C}}_{n}$, $C_{n}^{’}$, $D_{n}$, and $D_{n}^{’}$ are undetermined coefficients. In the spherical coordinate system, the magnetic vector potential outside the TM should have the following form

(3) $$\;\dot{A}=\frac{1}{2}{\dot{B}}_{e}rP_{1}^{1}(\cos\theta )e_{\varphi }$$

By comparing the second equation of Equation (2) with that of Equation (3), it can be seen that Equation (2) can be satisfied only when *n* = 1. Since $\dot{A}$ has finite values at *r* = 0, *r* = ∞, *θ* = 0, and *θ* = π, then ${\dot{C}}_{n}=B_{n}^{’}=A_{n}^{’}=D_{n}^{’}=0$. Based on the above boundary conditions, we can derive the following equation

(4) $$\dot{A}=\left\{\begin{array}{ll} \frac{1}{2}{\dot{B}}_{e}(r+\dot{C}r^{-2})\sin\theta e_{\varphi } & a<r<+\infty \\ \frac{1}{2}{\dot{B}}_{e}\dot{D}r^{-1/2}I_{3/2}(\lambda r)\sin\theta e_{\varphi } & 0<r<a \end{array}\right.$$

Further utilizing the boundary conditions on the interface between the TM and the vacuum environment where *r* = *a*, namely

(5) $$\left\{\begin{array}{l} {\left.\frac{\partial (\dot{A}\sin\theta )}{\partial \theta }\right|}_{r=a_{+}}={\left.\frac{\partial (\dot{A}\sin\theta )}{\partial \theta }\right|}_{r=a_{-}}\\ {\left.\frac{1}{\mu_{0}}\frac{\partial (r\dot{A})}{\partial r}\right|}_{r=a_{+}}={\left.\frac{1}{\mu }\frac{\partial (r\dot{A})}{\partial r}\right|}_{r=a_{-}} \end{array}\right.$$

We can get the expressions of $\dot{C}$ and $\dot{D}$ as follows

(6) $$\left\{\begin{array}{l} \dot{C}=a^{3}\frac{(2\mu +\mu_{0})\lambda aI_{-1/2}(\lambda a)-[\mu_{0}(1+\lambda^{2}a^{2})+2\mu ]I_{1/2}(\lambda a)}{\left(\mu -\mu_{0})\lambda aI_{-1/2}(\lambda a)+[\mu_{0}(1+\lambda^{2}a^{2})-\mu ]I_{1/2}(\lambda a\right)}\\ \dot{D}=\frac{3\mu \lambda a^{5/2}}{\left(\mu -\mu_{0})\lambda aI_{-1/2}(\lambda a)+[\mu_{0}(1+\lambda^{2}a^{2})-\mu ]I_{1/2}(\lambda a\right)} \end{array}\right.$$

According to the law of electromagnetic induction, the analytical expression of the induced eddy current within the TM can be obtained, that is

(7) $${\dot{i}}_{\varphi }=-\sigma \frac{\partial {\dot{A}}_{\varphi }}{\partial t}=-\frac{1}{2}j\omega \sigma {\dot{B}}_{e}\dot{D}r^{-1/2}I_{3/2}(\lambda r)\sin\theta e_{\varphi }$$

According to the definition of eddy current loss, the average loss power of the sphere TM in one cycle is

(8) $$\begin{array}{cc} P_{eddy} & =-\frac{1}{2}\int_{V}\frac{{\dot{i}}_{\varphi }\cdot {\dot{i}}_{\varphi }^{*}}{\sigma }dV'\\ & =-\frac{\pi \sigma \omega^{2}B_{0}^{2}}{3}{\dot{D}}^{2}\int_{0}^{a}I_{3/2}(\lambda r)\cdot \;I_{3/2}(j\lambda r)rdr \end{array}$$

where *B*_0_ is the amplitude of${\;\dot{B}}_{e}$. After complex mathematical derivations (see the Appendix A for details), the expression for *P_eddy_* of the spherical TM can be simplified as

(9) $$P_{eddy}=-\frac{1}{15}\pi a^{5}\sigma \omega^{2}B_{0}^{2}$$

### 2.2. The Equivalent Process of Eddy Current Formation

**Figure 2:**
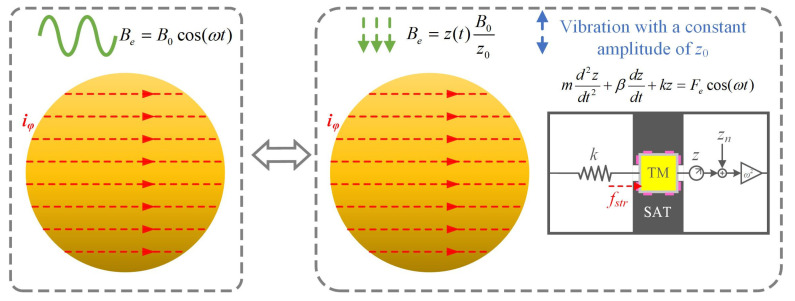
Schematic diagram of the equivalent process of eddy current formation.

The formulation of the eddy current is that the AUMF with angular frequency of *ω* acts on the TM, as shown in the left diagram of Figure 2. This formulation process can equal the following process: regarding the TM as an oscillator, which makes simple harmonic vibration in the *z*-direction with a constant amplitude of *z*_0_ and angular frequency *ω* in a magnetic field with a constant gradient of ∂*B*/∂*z*, like a spring-damping system, as shown in the right diagram of Figure 2. This equivalent process can be expressed by the following differential equation

(10) $$m\frac{d^{2}z(t)}{dt^{2}}+\beta \frac{dz(t)}{dt}+\kappa z(t)=F_{e}\cos(\omega t)$$

where *z*(*t*) is the displacement of the oscillator at *t*, *β* is the damping coefficient, *κ* is the stiffness coefficient, and *F_e_* is the applied external force, which maintains the oscillator to perform simple harmonic motion with equal amplitude. When the system reaches the steady state, the solution of Equation (10) is

(11) $$z(t)=z_{0}\cos(\omega t-\varphi_{0})$$

where *φ*_0_ means the initial phase, which can be set as 0. At this moment, the velocity of the oscillator can be expressed as

(12) $$v_{z}(t)=-z_{0}\omega \sin(\omega t)$$

The magnetic field where the TM is located also changes in accordance with the law of simple harmonic vibration, and can be expressed as

(13) $$\begin{array}{cc} B_{e}(t) & =z(t)\cdot \frac{\partial B}{\partial z}\\ & =z_{0}\cdot \frac{\partial B}{\partial z}\cos(\omega t) \end{array}$$

Therefore, the amplitude of the magnetic field is

(14) $$B_{0}=z_{0}\frac{\partial B_{e}}{\partial z}$$

By substituting Equation (14) into Equation (9), it can be obtained that

(15) $$P_{eddy}=-\frac{1}{15}\pi a^{5}\sigma {\left(\frac{\partial B}{\partial z}\right)}^{2}{\left(z_{0}\omega \right)}^{2}$$

### 2.3. Loss Equivalence and Damping Coefficient

In the aforementioned process of simple harmonic vibration with equal amplitudes, due to the existence of damping, the average power consumed by the system within one cycle is

(16) $$\begin{array}{cc} P_{damp} & =\frac{1}{T}\int_{0}^{T}\beta z(t)\cdot v_{z}(t)dt\\ & =-\frac{\omega^{2}\beta z_{0}^{2}}{2\pi }\int_{0}^{\frac{2\pi }{\omega }}\cos(\omega t-\varphi_{0})\cdot \sin(\omega t-\varphi_{0})dt\\ & =-\frac{\beta \omega^{2}z_{0}^{2}}{2} \end{array}$$

In the simple harmonic vibration, the mechanical energy of the system is dissipated in the form of damping loss. Since the eddy current formation process has been made equivalent to a spring-damping system that undergoes equal-amplitude oscillation, the eddy current loss is equal to the damping loss, that is

(17) $$P_{damp}=P_{eddy}$$

Substituting Equation (15) and Equation (16) into Equation (17), we can obtain the damping coefficient

(18) $$\beta =\frac{2}{15}\pi a^{5}\sigma {\left(\frac{\partial B}{\partial z}\right)}^{2}$$

### 2.4. The ECDMAN

For a damped harmonic oscillator system, considering the influence of the elastic restoring force, if its motion is affected by a random force *F*(*t*), the Langevin equation that describes Brownian motion can be expressed as

(19) $$m\frac{d^{2}x}{dt^{2}}+\beta \frac{dx}{dt}+kx=F(t)$$

In this equation, *m* represents the mass of the harmonic oscillator, *x* is the displacement, *k* is the stiffness coefficient, and *β* denotes the damping coefficient, which describes the characteristics of energy dissipation caused by the interaction between the system and its surrounding environment. By comparing Equation (10) with Equation (19), it can be seen that the damped simple harmonic vibration is a special form of Brownian motion. According to the standard fluctuation-dissipation formula [24], the power spectral density of the thermal noise in Brownian motion is

(20) $$S(\omega )=4k_{B}T\beta$$

The energy of the eddy current in the TM will eventually be dissipated in the surrounding environment in the form of thermal energy. In the system composed of the TM and its surrounding environment, a thermal equilibrium state will eventually be reached. In Section 2.2 and Section 2.3, we regarded the formation process of the eddy current under the influence of the AUMF as being equivalent to the process where the oscillator performs simple harmonic vibrations having a constant amplitude in a constant gradient magnetic field. Consequently, the eddy current loss is equal to the damping loss of the simple harmonic vibration, and we then obtained the expression for the damping coefficient β, which is Equation (18). By substituting Equation (18) into Equation (20), the PSD of the thermal noise introduced by the eddy current dissipation can be obtained, that is

(21) $$S_{eddy}=\frac{8}{15}k_{B}T_{EH}\pi a^{5}\sigma {\left(\frac{\partial B}{\partial z}\right)}^{2}$$

According to the relationship between amplitude spectral density (ASD) and PSD, the ASD of the thermal noise is

(22) $$\begin{array}{cc} A_{eddy} & =\sqrt{S_{eddy}}\\ & =\sqrt{\frac{8}{15}k_{B}T_{EH}\pi a^{5}\sigma {\left(\frac{\partial B}{\partial z}\right)}^{2}} \end{array}$$

From the perspective of the Langevin equation, it is the random force that serves as the source generating thermal noise. Consequently, the amplitude spectral density (ASD) of the acceleration noise, which is introduced by the thermal fluctuations of the eddy current and is defined as the ECDMAN in this paper, can be expressed as follows

(23) $$\begin{array}{cc} \delta a_{eddy} & =\frac{1}{m_{TM}}A_{eddy}\\ & =\frac{1}{m_{TM}}\frac{\partial B}{\partial z}\sqrt{\frac{8\pi }{15}k_{B}T_{EH}a^{5}\sigma } \end{array}$$

where *k_B_* is the Boltzmann constant, *T_EH_* represents the temperature within the electrode house, and *m*_TM_ represents the mass of the TM. If the side length of the cubic TM is *L*, then the radius of the spherical TM with equal volume is

(24) $$a=\sqrt[{3}]{\frac{3}{4\pi }}L$$

Substituting Equation (24) into Equation (23), one can obtain the model of ECDMAN, namely

(25) $$\delta a_{eddy}=\frac{{\left(3/\pi \right)}^{1/3}}{2^{1/6}m_{TM}}\sqrt{\frac{k_{B}T_{EH}\sigma L^{5}}{5}}\cdot \frac{\partial B}{\partial z}$$

## 3. Simulation Result

In this paper, three key conclusions, namely the model of the spherical TM’s eddy current loss Equation (9), the model of the equivalent process of eddy current formation Equations (15) and (17), which represents the equality of damping loss and eddy current loss, are verified by FEM software. The process of obtaining MAN based on the damping coefficient *β* adopts the classic fluctuation-dissipation theorem, and no further verification is required. In the following simulations, two types of FEM software are used. One is Comsol Multiphysics 6.1 and the other is Altair Flux 2020.1, which has advantages in terms of the kinematics simulation driven by electromagnetic forces. A mesh independence test was conducted in the simulation. The Comsol FEM model consists of a total of 293,815 volume elements and 52,342 nodes, and the Flux FEM model consists of a total of 136,514 volume elements and 23,644 nodes. The AUMF and the constant gradient magnetic field are both set in the background magnetic field interface. The relevant parameters of the TM published by LPF are adopted in the simulations (Reference [7]), as shown in Table 1.

### 3.1. Simulation of the Eddy Current Loss Model of the Spherical TM

**Figure 3:**
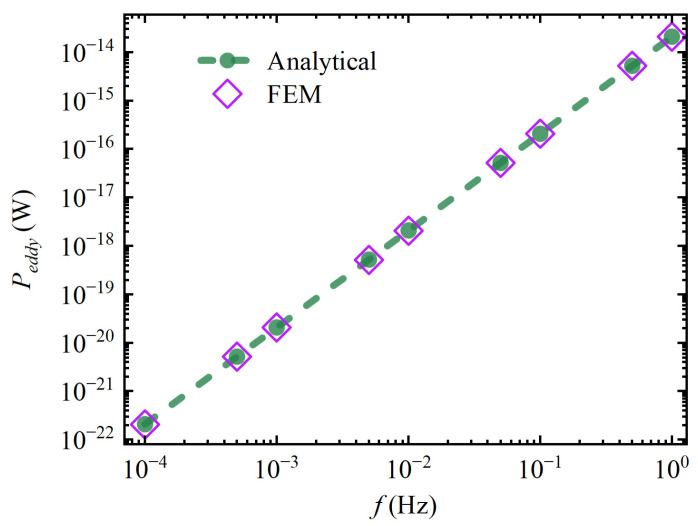
The eddy current loss of a spherical TM.

Equation (9) is simulated and verified using the FEM software Comsol Multiphysics, and the results are shown in Figure 3. It can be seen that in the target frequency band range from 10⁻⁴ to 1 Hz, the calculation results of the analytical method and the FEM are essentially consistent, and the maximum relative deviation between them does not exceed 0.31%. This proves the correctness of the analytical model Equation (9).

### 3.2. Simulation of the Equivalent Process of Eddy Current Formation

**Figure 4:**
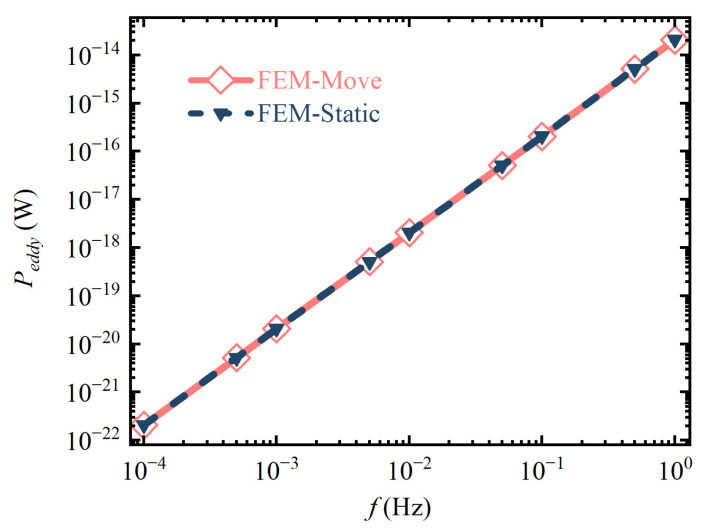
The simulation result of the two processes of the generation of eddy current loss.

Figure 4 shows the simulation result of the loss equivalent model by the FEM software. The dark blue triangular dotted line is the eddy current loss curve of the spherical TM under the action of AUMF, and the red rhombus solid line is the eddy current loss curve generated by the spherical TM performing equal-amplitude simple harmonic motion under a uniform gradient magnetic field. It can be seen that the two curves are essentially coincident, and the maximum relative deviation between them in the target frequency band is 0.99%, which verifies the correctness of the loss equivalent model Equation (15).

### 3.3. Simulation of the Equivalence of Damping Loss and Eddy Current Loss

**Figure 5:**
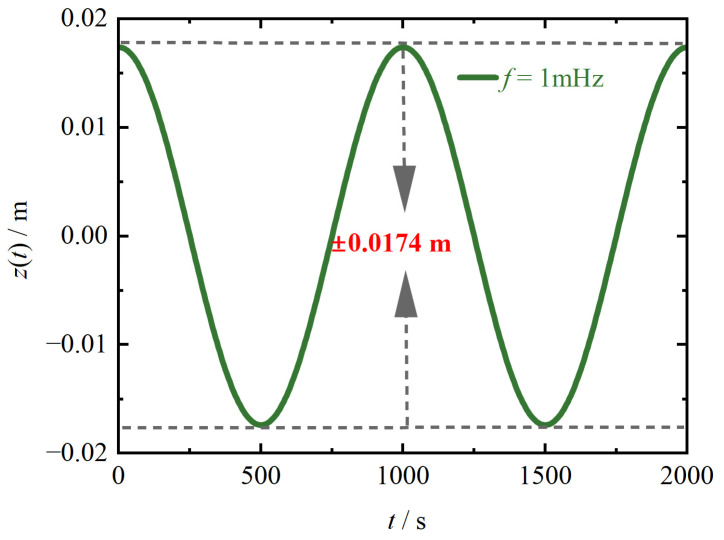
The displacement curve of the TM.

The transient module within the FEM software Altair Flux is employed to conduct the simulation of Equation (17). Additionally, within the mechanical motion interface, the values of the initial velocity, harmonic force, stiffness coefficient, and damping coefficient corresponding to the TM are assigned. Then, the average damping loss of the TM at each frequency point is calculated and compared with the eddy current loss. Figure 5 takes the frequency of 1 mHz as an example and shows the z-direction displacement of the TM within two periods obtained by FEM software. It can be seen that the TM performs simple harmonic motion with equal amplitude within the range of ±1.74 cm.

**Figure 6:**
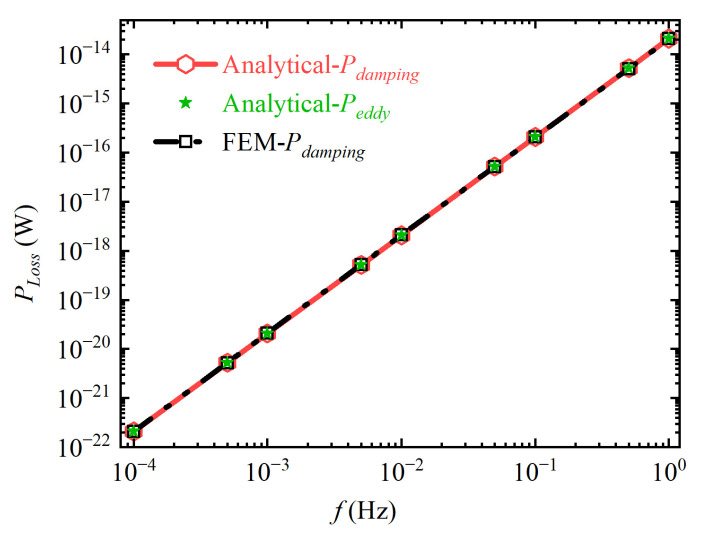
Simulation result of the equivalence of damping loss and eddy current loss.

The damping losses obtained by Equation (16) and FEM are shown as the red hexagonal solid line and the black quadrilateral dotted line in Figure 6, respectively. The two curves basically coincide, and the maximum relative deviation between the damping loss obtained by the analytical method and that obtained by the FEM is 2.42%. This proves the correctness of Equation (16). In addition, the green pentagram represents the eddy current loss at each frequency point. It is essentially consistent with the damping loss, and the maximum relative deviation between them is 2.36%. This proves the correctness of Equation (17).

## 4. Evaluation and Modification of the ECDMAN

### 4.1. Evaluation of the ECDMAN

**Figure 7:**
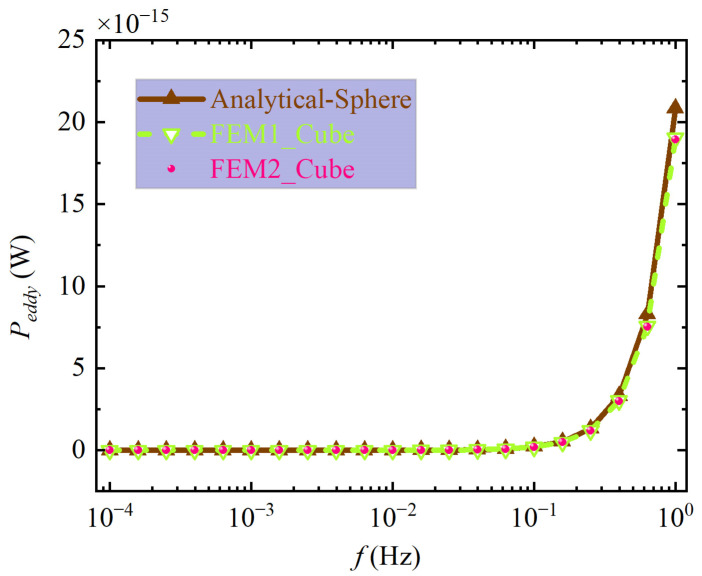
Comparison of eddy current loss between spherical TM and cubic TM.

With the parameters given in Table 1, the MAN introduced by the eddy current dissipation is calculated to be approximately 1.23 × 10^−16^ m/s^2^/Hz^1/2^ according to Equation (25). However, Equation (25) is essentially the ECDMAM model for the spherical TM, so this value is still the evaluation result of the spherical TM’s ECDMAM. Due to the influence of the conductivity, permeability, size of the TM, and the frequency of the environmental magnetic field, the eddy current losses of the cubic TM and the spherical TM are not exactly equal. Figure 7 shows the eddy current losses of the spherical TM calculated by the analytical method and result of the cubic TM’s eddy current losses calculated by two kinds of FEM software. The correctness of the analytical method for calculating the eddy current loss of the spherical TM has been verified in Section 3.1. For this reason, only Comsol Multiphysics (FEM1) and Altair Flux (FEM2) are used to calculate the eddy current loss of the cubic TM here, as shown by the green dashed line and magenta dots in the figure, respectively. The maximum deviation of FEM1 relative to FEM2 is 0.48%. The two FEMs verify each other, indicating the correctness of their calculation results. It can be seen from Figure 7 that the eddy current loss of the spherical TM is larger than that of the cubic TM, and their deviation gradually increases with the increase in frequency.

### 4.2. Modification of ECDMAN

**Figure 8:**
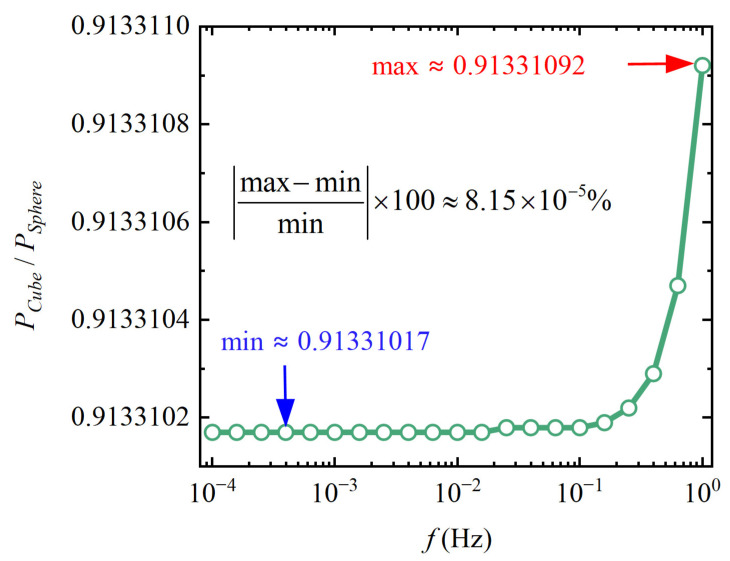
The ratio of the eddy current loss of the cubic TM to the spherical TM.

Figure 8 shows the ratio of the eddy current loss of the cubic TM to that of the spherical TM

(26) $$C_{coef}(f)=\frac{P_{eddy}^{Cube}}{P_{eddy}^{Sphere}}$$

The ratio *C_coef_* is a function of *f*. However, within the target frequency band, the maximum *C_coef_* is 0.91331092, and the minimum *C_coef_* is 0.91331017. The deviation of the maximum relative to the minimum is about 8.15 × 10^−5^%, which is extremely small. Hence, the average value of *C_coef_*, which is approximately 0.91331023 over the entire frequency band, is taken as the modification coefficient for the eddy current loss. For the TM shown in Table 1, the modified expression of ECDMAN is

(27) $$\delta a_{modi-eddy}=\frac{{\left(3/\pi \right)}^{1/3}}{2^{1/6}m_{TM}}\sqrt{\frac{C_{coef}k_{B}T_{EH}\sigma L^{5}}{5}}\cdot \frac{\partial B}{\partial z}$$

After correction, the evaluation result of ECDMAN by Equation (27) is 1.18 × 10^−16^ m/s^2^/Hz^1/2^. Taking the result of the FEM as the criterion, the calculation error is reduced by approximately 4.64% comparing Equation (27) with Equation (25).

## 5. Discussion

**Figure 9:**
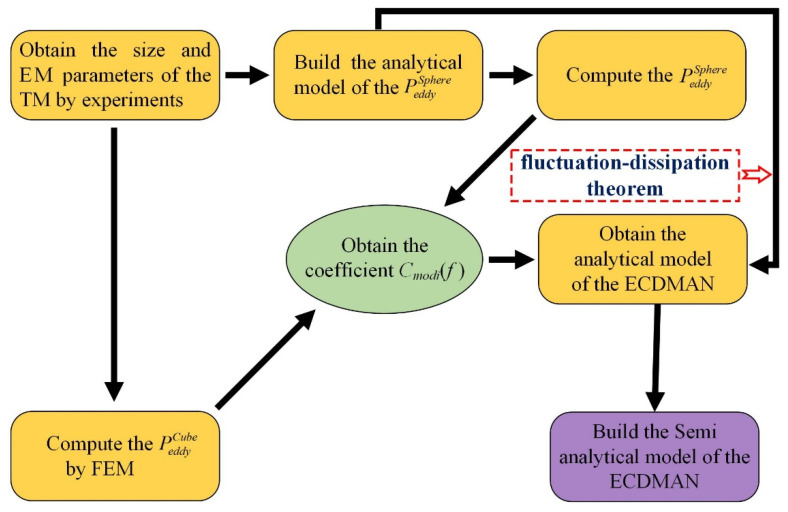
A generalized process for establishing the semi-analytical model of ECDMAN.

In this paper, an in-depth analytical exploration into the eddy current loss of the spherical TM is undertaken, with the objective of obtaining the analytical model of ECDMAN. Subsequently, the finite element method (FEM) is applied to compute the eddy current loss of the cubic TM. The ratio of the eddy current loss of the cubic TM to that of the spherical TM is used as the modification coefficient *C_coef_* to modify the analytical model of ECDMAN, thereby obtaining the semi-analytical model of ECDMAN. Strictly speaking, *C_coef_* is not only related to the frequency. However, it is also a function of conductivity, susceptibility, and the size of the TM, and the accurate expression of *C_coef_* is therefore difficult to obtain. In the context of practical engineering, the aforementioned parameters can be measured via ground-based experiments or in-orbit experiments. Meanwhile, the relationship between the modification coefficient *C_coef_* and the frequency can be derived through the methodology described within this paper. By following such procedures, a generalized semi-analytical model of ECDMAN can be obtained. Moreover, in future in-orbit experiments, the rapid and accurate evaluation of this noise term will be realized. The flowchart for the approach of establishing the semi-analytical model is summarized in Figure 9.

In addition, the total MAN of the inertial sensor of the LPF is approximately 1.55 × 10^−15^ m/s^2^/Hz1/2, and the ECDMAN accounts for about 7.61% of the total MAN. Although this is not a significant value, it is still of significance for ECDMAN.

Firstly, in the vicinity of the TM, there are 24 negative temperature coefficient (NTC) thermistors [22]. These thermistors incorporate magnetic materials and thereby introduce relatively substantial gradients as well as gradient fluctuations into the environment surrounding the TM. Consequently, the magnetic acceleration noise based on the principle of the force acting on a magnetic dipole in a magnetic field (MDMAN) dominates. Nevertheless, in future gravitational wave detection missions, either the NTC thermistors will not be employed or thermal sensors founded on other principles will be adopted instead. As a consequence of this, the proportion of MDMAN will decrease significantly, whereas the proportion of ECDMAN will experience a further increase.

Secondly, the analysis model of ECDMAN involves parameters such as the conductivity *σ* and side length *L* of TM, the temperature *T_EH_* inside the vacuum chamber, and the gradient of the environmental magnetic field ∂*B*/∂*z*. Therefore, the evaluation result of ECDMAN is a boundary condition for the design of the TM, the vacuum chamber, and the satellite’s magnetic payload.

Finally, notwithstanding that the proportion of ECDMAN is not prominent, it still constitutes a component within the noise index system of the inertial sensor. Moreover, the noise index system plays a fundamental role and acts as the basis for the design of the inertial sensor.

## 6. Conclusions

Based on the principles of electromagnetism and the fluctuation-dissipation theorem, this paper conducts a systematic derivation and establishes an analytical model of ECDMAN corresponding to the spherical TM. This model can be used as an approximate model to evaluate the ECDMAN of the cubic TM. By taking the TM of the LPF as an illustrative example, the modification coefficient of ECDMAN is acquired through the combination of the analytical method and the FEM. Subsequently, a semi-analytical model of ECDMAN for the cubic TM is established. Compared to the approximate model, the calculation error of the semi-analytical model decreased by approximately 4.64%. On this basis, a generalized modeling approach for the semi-analytical model of ECDMAN is put forward. This approach is applicable to TMs featuring different parameters and enables the real-time and rapid evaluation of ECDMAN during in-orbit experiments. The ECDMAN studied in this paper is an indispensable part of the MAN of the space inertial sensor, and is of great significance for guiding its parameter design and constructing the noise index system.

## Figures and Tables

**Table 1:** The parameter list adopted in the FEM simulations.

Parameter Name	Value
Length of the TM	46 mm
Susceptibility of the TM	2.5 × 10^−5^
Conductivity of the TM	3.33 × 10^6^ S/m
Mass of the TM	1.96 kg
Amplitude of the AUMF	200 × 10^−9^ T
Constant magnetic field gradient	11,500 × 10^−9^ T/m
Stiffness coefficient	3 N/m
Temperature of the electrode house	303 K

## Data Availability

The data are contained within the article.

## Appendix A

The derivation from Equation (8) to Equation (9) is as follows: the analytical expressions of the modified Bessel functions of order 3/2, 1/2, and −1/2 are

(A1) $$I_{3/2}(x)=\sqrt{\frac{2}{\pi x}}[\cosh(x)-\frac{1}{x}\sinh(x)]$$

(A2) $$I_{1/2}(x)=\sqrt{\frac{2}{\pi x}}\sinh(x)$$

(A3) $$I_{-1/2}(x)=\sqrt{\frac{2}{\pi x}}\cosh(x)$$

Substituting Equation (A1) into Equation (8), we can get that

(A4) $$\begin{array}{c} \int_{0}^{a}I_{3/2}(\lambda r)\cdot \;I_{3/2}(j\lambda r)rdr\\ =\frac{\sqrt{-j}\{\lambda a\cosh(\lambda a)\sin(\lambda a)+[\lambda a\cos(\lambda a)-2\sin(\lambda a)]\sinh(\lambda a)\}}{\pi \lambda^{3}a} \end{array}$$

Let *λa* = *k*, then there is

(A5) $$D^{2}=\frac{9\pi a^{3}k^{3}\mu^{2}}{2{\left[k(\mu -\mu_{0})\cosh(k)+(\mu_{0}-\mu +\mu_{0}k^{2})\sinh(k)\right]}^{2}}$$

As *λa* is a minimum value, then *k*→0. It can be obtained that

(A6) $$\begin{array}{c} \underset{k\to 0}{\lim}{\dot{D}}^{2}\int_{0}^{a}I_{3/2}(\lambda r)\cdot \;I_{3/2}(j\lambda r)rdr\\ =-\frac{9(1-i)a^{5}\mu^{2}}{5\sqrt{2}{\left(\mu +2\mu_{0}\right)}^{2}} \end{array}$$

Similarly, since the susceptibility of TM is an extremely small value, Equation (A6) can be simplified as

(A7) $$\begin{array}{c} {\dot{D}}^{2}\int_{0}^{a}I_{3/2}(\lambda r)\cdot \;I_{3/2}(j\lambda r)rdr\\ \approx -\frac{1-j}{5\sqrt{2}}a^{5} \end{array}$$

Then, Equation (9) can be derived by substituting the Equation (A7) into Equation (8) and taking its modulus value.
